# Emergent Self-Assembly of Sustainable Plastics Based on Amino Acid Nanocrystals

**DOI:** 10.1021/acsnano.3c02528

**Published:** 2023-10-23

**Authors:** Angelica Niazov-Elkan, Haim Weissman, Eyal Shimoni, XiaoMeng Sui, Yishay Feldman, H. Daniel Wagner, Boris Rybtchinski

**Affiliations:** † Department of Molecular Chemistry and Materials Science, Weizmann Institute of Science, Rehovot 76100, Israel; ‡ Department of Chemical Research Support, Weizmann Institute of Science, Rehovot 76100, Israel

**Keywords:** Sustainable plastic, self-assembly, sustainable composite, crystal growth, tyrosine

## Abstract

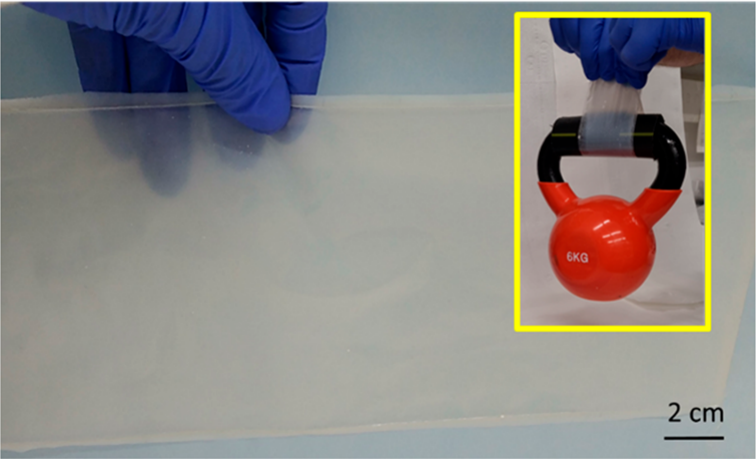

Development of biodegradable plastic materials is of primary importance in view of acute environmental and health problems associated with the accumulation of plastic waste. We fabricated a biodegradable composite material based on hydroxyethyl cellulose polymer and tyrosine nanocrystals, which demonstrates enhanced strength and ductility (typically mutually excluding properties), superior to most biodegradable plastics. This emergent behavior results from an assembly pattern that leads to a uniform nanoscale morphology and strong interactions between the components. Water-resistant biodegradable composites encapsulated with hydrophobic polycaprolactone as a protection layer were also fabricated. Self-assembly of robust sustainable plastics with emergent properties by using readily available building blocks provides a valuable toolbox for creating sustainable materials.

## Introduction

Polymer-based plastic materials accumulate in marine environments (Great Pacific Garbage Patch),^1^ land,^2^ and as microplastics, in drinking water,^3^ and air,^4^ resulting in acute environmental and health problems.^5^ This is because most plastics are not biodegradable, often being discarded as waste, while recycled plastic constitutes only 9–18% of the plastic matter ever produced.^2,5^ There is an ongoing effort to develop biodegradable plastics, but the limited range of available materials and their properties hinder wide utilization.^6^

Material strength and ductility (plastic strain) are usually mutually exclusive mechanical properties. One of the key challenges in materials science is to resolve the conflict of simultaneously attaining strength and ductility.^7,8^ Biological materials often demonstrate high strength and ductility, owing to advantageous arrangement of rigid and soft components.^9−12^ A prime example of outstanding mechanical properties is represented by silk where intricate arrangement of rigid crystalline and softer amorphous protein regions results in a superbly strong material that is entirely organic;^11^ however, emulating these attributes in artificial materials has proven to be extremely challenging.

Some crystalline organic materials based on biomolecules exhibit high Young’s moduli.^13^ We envisaged that interfacing such biomolecular crystals with biodegradable polymers could lead to biodegradable materials with superior mechanical properties. In this respect, organic crystals offer distinct advantages: they may have high affinity to the polymer matrix and, in the case of nanocrystals, allow nanostructured morphology advantageous to mechanical properties. Importantly, the organic nanocrystals based on small molecules can be grown directly within the polymer matrix, unlike conventional rigid fillers. Furthermore, biomolecular crystal/polymer self-assembly can be performed in aqueous media, thus enabling sustainable fabrication and advantageous assembly modes.^14,15^

**Figure 1:**
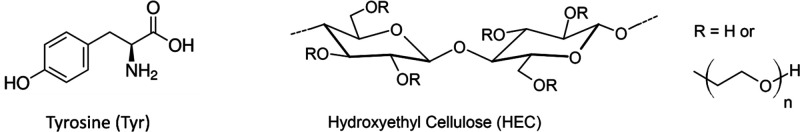
Molecular structure of Tyr and HEC.

Herein, we report on the fabrication of a biodegradable composite material based on a hydroxyethyl cellulose (HEC) polymer and tyrosine (Tyr) nanocrystals (Figure 1). This material demonstrates enhancement in modulus, strength, and ductility (strain), superior to most biodegradable plastics. The emergent behavior is achieved by a specific assembly pattern, resulting in strong Tyr/HEC interactions and a distinctive nanoscale morphology. Self-assembly of robust sustainable plastics with emergent properties using readily available building blocks provides a valuable toolbox for creating sustainable materials.

## Results and Discussion

Our material design concept was based on the idea that linear, flexible polysaccharide chains may interact favorably with Tyr crystals that exhibit high Young’s modulus (20–40 GPa^16^), especially if these crystals were grown within the polymer matrix. The ductile polysaccharide combined with the stiff Tyr crystals could effectively result in reinforcement of the composite structure. We chose alginate, agarose, and hydroxyethyl cellulose (HEC) as polymer matrices, based on their high ductility, solubility in aqueous medium, cost, availability, biodegradability, and biocompatibility. We observed no enhancement in mechanical properties when using alginate, a modest improvement with agarose (Figure S1), and a substantial enhancement with HEC (as discussed below).

**Figure 2:**
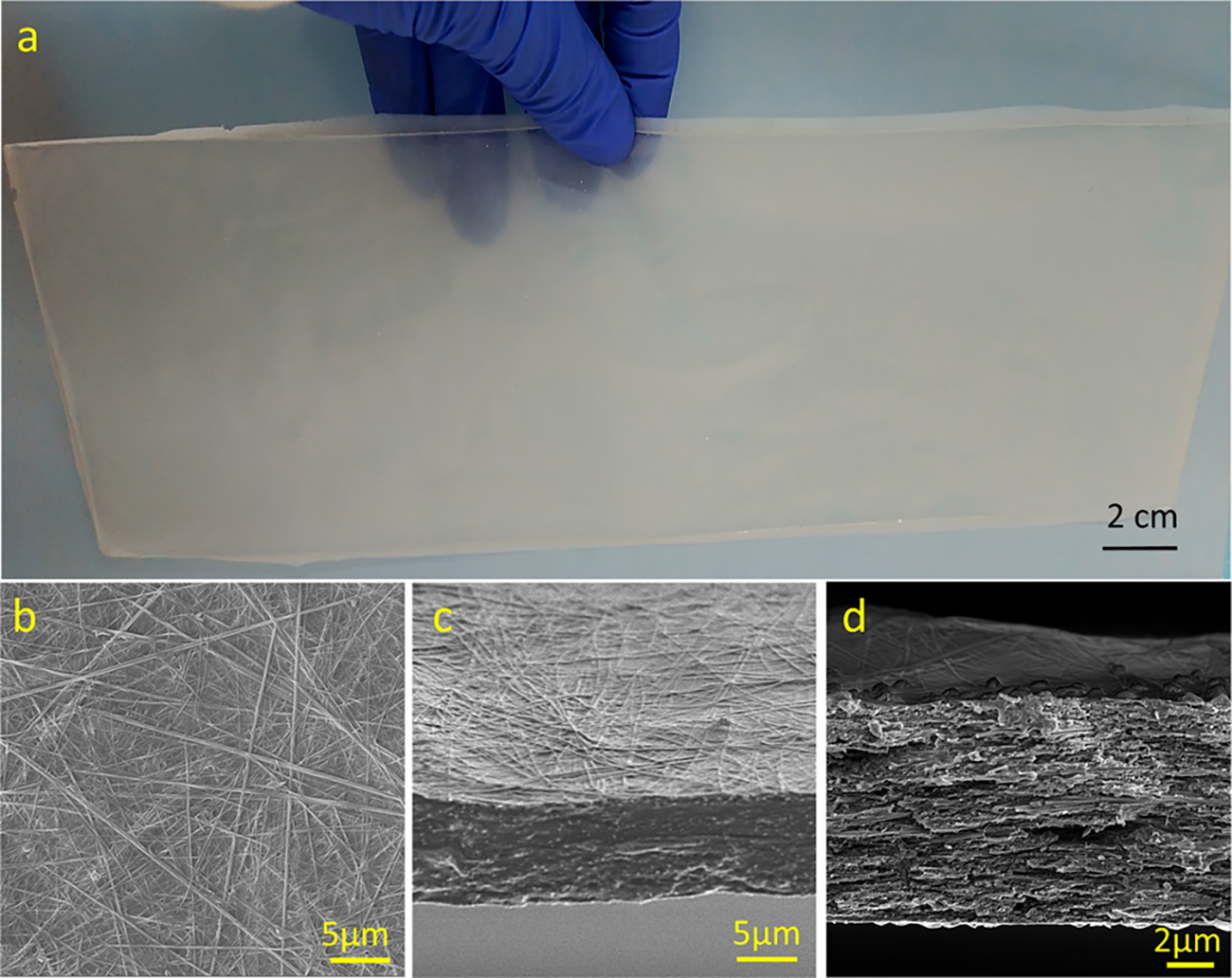
HEC/Tyr (10:3, wt %) film. (a) A photograph of the film. (a–c) SEM images: (b) top view; (c) cross section; (d) zoomed-in image of the cross section.

**Figure 3:**
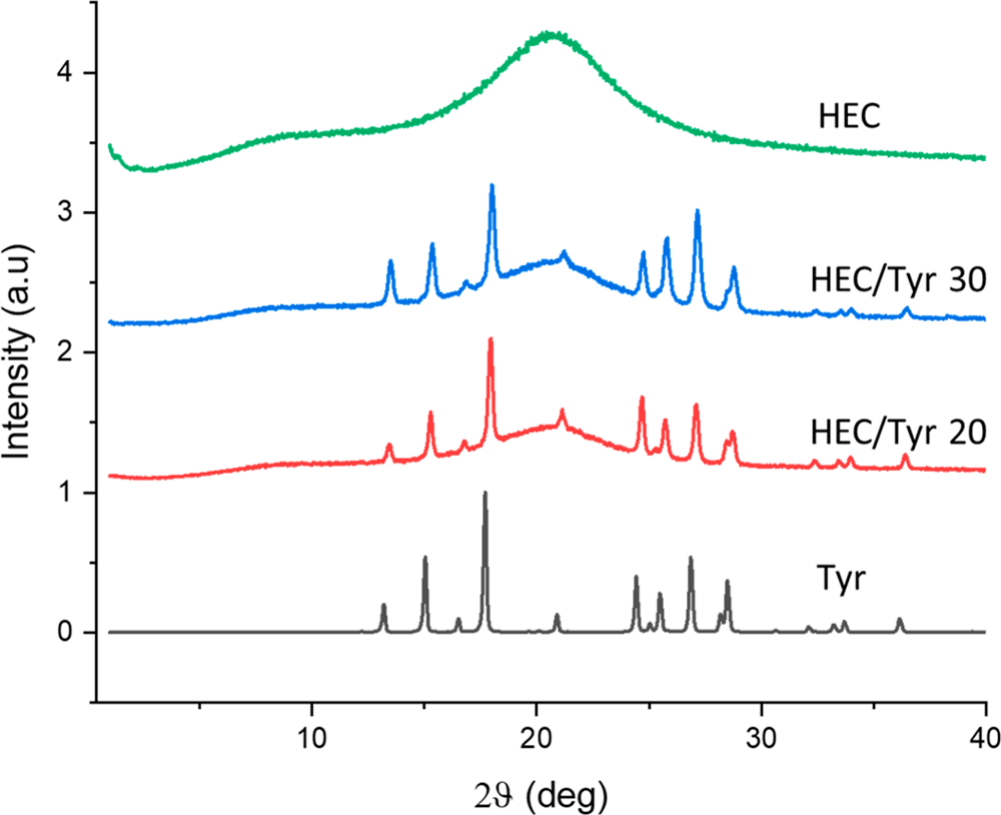
XRD diffractograms of Tyr crystals, HEC, and HEC/Tyr hybrids.

To fabricate a hybrid material, we interfaced Tyr nanocrystals with an HEC polymer (Figure 1). HEC, a ductile linear polymer obtained by chemical modification of native cellulose, serves as a gelling and thickening agent, widely used in personal care products, coatings, pharmaceuticals, and adhesives.^17^ The fabrication of the HEC/Tyr composite was carried out in aqueous solution (see the Experimental Section). Briefly, Tyr dissolved in boiling water was added to an aqueous solution of HEC, and the resulting mixture was stirred at ambient temperature, becoming opaque after several hours due to Tyr crystallization. The mixture was cast onto either a Petri dish or a rectangular mold to afford solid semitransparent films, 20–50 μm in thickness, after drying overnight at ambient conditions (Figure 2a). Optical microscopy and SEM images (Figure 2) of the resultant HEC/Tyr films showed a uniform distribution of the Tyr crystals within the polymer matrix. The films retained 8–10% water by weight, as indicated by TGA (Figure S2), with this content remaining constant at ambient conditions for months. The presence of Tyr crystals in the composite was confirmed by XRD (Figure 3) and Raman spectroscopy (Figure S3), indicating that the crystals correspond to a known crystal structure of Tyr.^16^ XRD indicates that the Tyr crystals exhibit similar orientation as expected for needle-like crystals, which commonly exhibit parallel alignment with the substrate they are deposited onto. TGA, DSC, and XRD revealed that the films retain their structure up to 150 °C, except for the water loss (Figures S2, S4, S5).

**Figure 4:**
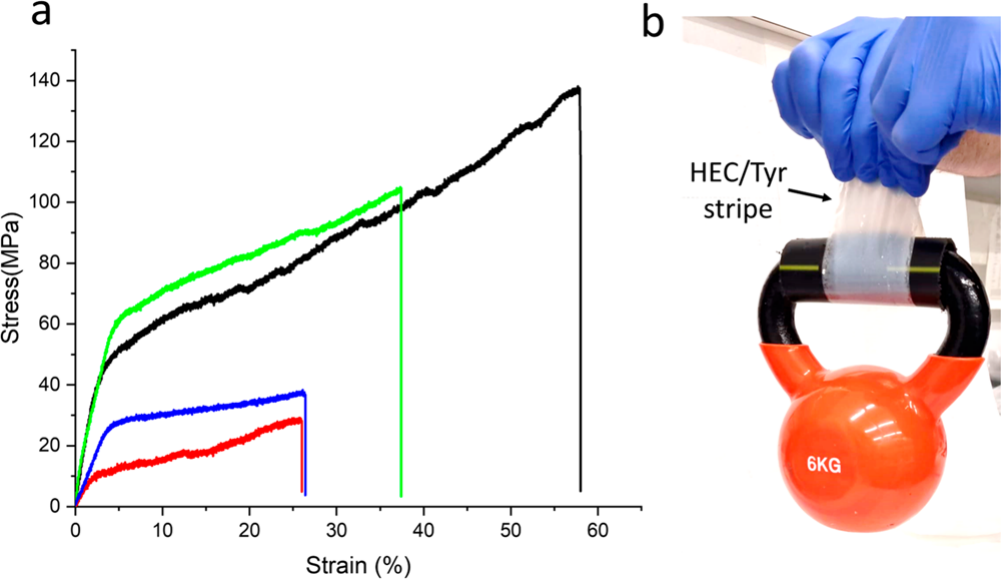
(a) Representative stress–strain curves of HEC (red) and HEC/Tyr hybrids: 10:2 (w/w, blue), 10:3 (w/w, black), 10:4 (w/w, green); (b) HEC/Tyr (10/3, w/w) hybrid stripe, 40-μm thick, lifting 6-kg weight.

HEC/Tyr composites exhibit a significant increase in modulus, strength, and elongation in comparison to pure HEC (Figure 4a, Table 1). For example, the HEC/Tyr composite with a composition of 10:3 wt % exhibited a 3-fold increase in modulus and 40% increase in strain (Figure 4a, Table 1), resulting in a 6-fold rise in toughness. Above 40 wt % Tyr, the HEC/Tyr mechanical properties deteriorated (Table 1, HEC:Tyr = 10:5 wt %) due to defects resulting from the high Tyr loading (Figure S6). To further exemplify the robustness of the composite, we conducted a weight-bearing test using 40-μm thick composite strips. These strips exhibited stability under a load of 6 kg without any noticeable deformation, underscoring the load-bearing capacity of the material (Figure 4b).

**Table 1:** Mechanical Properties of HEC and HEC/Tyr Hybrids

	Modulus (GPa)	Strength (MPa)	Strain (%)	Toughness (MPa)
**HEC**	0.44 ± 0.13	33 ± 16	32 ± 9	6 ± 3
**HEC/Tyr** **10:2**	0.83 ± 0.18	64 ± 18	29 ± 12	31 ± 14
**HEC/Tyr** **10:3**	1.26 ± 0.26	101 ± 22	55 ± 10	36 ± 12
**HEC/Tyr** **10:4**	2.09 ± 0.61	71 ± 19	48 ± 17	20 ± 11
**HEC/Tyr** **10:5**	1.37 ± 0.18	21 ± 3	4 ± 1	1 ± 0.2

In terms of enhanced strength and toughness, Tyr/HEC outperforms biodegradable or partially biodegradable plastics used for packaging, such as starch blends (including thermoplastic starch, TPS),^18^ Polylactic acid (PLA),^19,20^ Polyglycolic acid (PGA),^21^ PLA/PGA blends,^21,22^ TPS blends based on low density polyethylene (LDPE),^19,23^ PLA blends with high density polyethylene,^24^ and several other materials (Table S1). While stable at ambient humidity, HEC/Tyr slowly absorbs liquid water, giving rise to a gel-like material. In order to create a water-resistant material, HEC/Tyr was laminated with polycaprolactone (PCL), a soft synthetic biodegradable polymer.^25^ The resultant material showed mechanical properties comparable to those of the parent HEC/Tyr (Table S2), and resistance to water (Figures S7 and S8, and Table S3).

**Figure 5:**
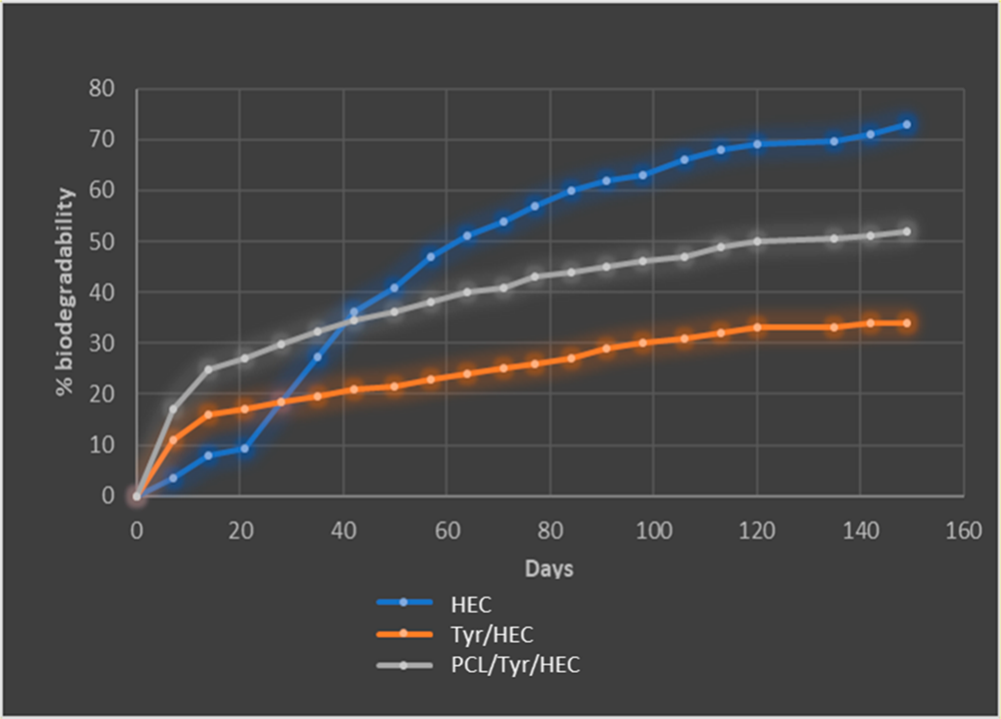
Biodegradation of the plastics.

HEC/Tyr and HEC/Tyr/PCL showed good biodegradability under standard conditions using the CO_2_ evolution test (see Experimental Section for details), losing 33 and 52 wt % respectively within 149 days (Figure 5). Further studies are planned in order to assess longer-term degradation of the systems.

The emergent mechanical properties of the Tyr/HEC arise from the specific assembly pattern resulting from the Tyr crystal growth within the HEC matrix. Thus, mixing the preformed Tyr nanocrystals with the HEC solution and subsequent drying resulted in an inhomogeneous material that had unsatisfactory mechanical properties.

**Figure 6:**
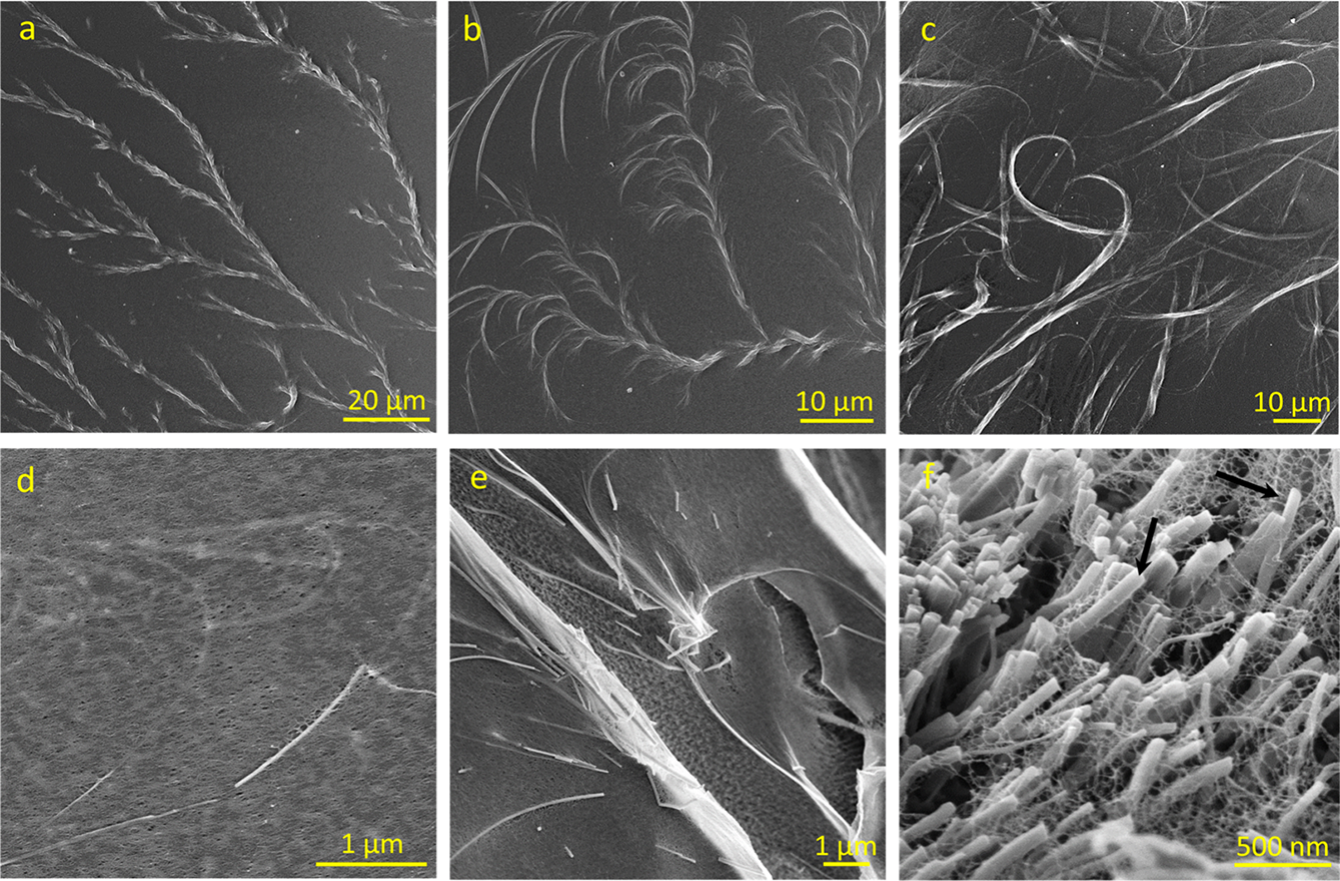
SEM (a–c) and cryo-SEM images (d–f) of Tyr crystal growth within aqueous HEC solution following addition of Tyr dissolved in hot water (final composition: HEC:Tyr = 10:3). (a) Immediately after mixing; (b) 30 min after mixing; (c) 1 h; (d) 90 min; (e–f) mature hybrid before water evaporation.

In order to obtain an insight into the assembly of the material, we performed SEM and cryo-SEM follow-up of the crystal growth within the polymer matrix (Figures 6 and S9–S13). While Tyr crystallizes into needle-like crystals from water, its crystallization within HEC solution gives rise to dendritic structures. Initially, thin needle-like Tyr crystallites form bundles that develop a fractal morphology (Figures 6a and S9). Subsequently, the crystals continue to grow, giving rise to larger stem-like bundles with branching structures (Figures 6b–d and S10, S11). Over time, this complex morphology evolves into a branched crystalline fibril network embedded within the HEC matrix (Figures 6f and S12, S13). The cryo-SEM image of the material at the end of the fabrication process revealed that the polymer chains envelop the uniformly dispersed Tyr nanocrystalline bundles, resulting in the interpenetrating HEC/Tyr network (Figures 6e,f and S13). Overall, Tyr crystal growth within the HEC polymer results in a branched network entrapped within polymer chains as a result of confinement imposed by the polymer matrix. The uniformity of the fibrillary network and its advantageous interaction pattern with HEC led to the synergy in terms of the mechanical properties: Young’s modulus increases owing to the rigid nature of the Tyr crystals, and the strain grows due the interpenetrating network, where the crystals contribute to the “unwinding” of HEC chains.^6^ Strong HEC/Tyr interactions were revealed by SEM imaging of the film cross sections following the tensile failure. It showed a sharp rupture pattern, featuring a uniform edge that indicates concurrent HEC and Tyr breakage, but also a slippage of the crystalline layers against each other, and the occasional protruding crystals, implying a pull-out mechanism accounting for high strain that leads to the tough material (Figure S14).

Evidently, wrapping of the HEC chains around the Tyr crystals (Figures 6f and S13) imposes significant mechanical interactions due to intimate polymer/crystal entanglement. These may be further augmented by noncovalent interactions, for example hydrogen bonding between the polymer chains and crystals. The latter could not be directly elucidated by Raman spectroscopy (Figure S3) due to multiple overlapping peaks, yet it is plausible based on the interaction mode observed in SEM (Figure 4). We further note that growing the Tyr crystals within the HEC matrix leads to the system where the crystalline and polymer networks must accommodate each other, as evidenced by the uniform Tyr crystal embedding pattern (Figures 6f and S13).

Strength/ductility trade-off precludes parallel increase of both. Typically, in polymer-based composites, reinforcing polymers with strong and stiff fillers such as glass and carbon fibers, clays, and nanocrystalline cellulose (NCC) yields composites with enhanced strength, but with lower strain (related to ductility) and toughness due to defect formation and other factors.^26^ Enhancing both strength and ductility can be achieved via precise control over the nanoscale structure, components distribution, and interaction patterns, as realized in biological materials such as bone, nacre, and silk.^9−12^ Tyr crystal growth within a polymer matrix allows optimal interactions, minimizing the defects and eventually forming a crystal network superbly entangled with the polymer matrix. The larger crystalline bundles that branch fibrous crystals represent additional cross-links within the system.

## Conclusions

We present a useful paradigm for sustainable plastic materials, employing a soft polymer matrix with biomolecular crystals grown within it, giving rise to both strong and ductile biodegradable plastic materials. Growing crystals within a polymer matrix affords an advantageous structure: the uniform crystalline network interwoven with the polymer one, leading to emergent mechanical properties represented by increase in modulus, strength, and strain. Water-resistant composites, Tyr/HEC encapsulated with hydrophobic PCL as a protection layer, were also fabricated, retaining biodegradability and enhanced mechanical performance. Emergent self-assembly of biodegradable plastic materials from readily available biomolecular building blocks was enabled by a simple crystallization process in an aqueous medium, conceptually advancing the development of sustainable plastics.

## Experimental Section

Solvents and reagents were purchased from commercial sources and used as received unless otherwise indicated. For all aqueous mixtures, double-distilled water (DDW) was used (Barnstead NANOpure Diamond water system). Organic solvents for spectroscopic and microscopic studies were of HPLC grade.

Sodium alginate (viscosity of 2% solution ∼250 cps, A2158), agarose (low gelling temp. BioReagent, A9414), HEC (2-hydroxyethyl cellulose, Mw = 1 × 10^6^ g·mol^–1^), l-Tyrosine, and Polycaprolactone (Mw = 8.4 × 10^4^ g·mol^–1^) were purchased from Sigma-Aldrich.

**Scanning electron microscopy (SEM) imaging** was performed using a Zeiss Supra 55 FEG-SEM or Ziess Ultra 55 FEG-SEM operating at 1–20 kV. Images were obtained using a working distance (WD) of 3–5 mm and a standard aperture (30 μm).

**Cryo-SEM** sample preparation involved a high-pressure freezing (HPF) technique. The samples were cut using a razor blade to fit in an aluminum disc (outer diameter 3.0 mm, thickness 0.5 mm, inner diameter 2.0 mm, depth 0.2 mm). 1-Hexadecane was used for filling empty space, and the disc was sealed with a flat disc. HPF was carried out using a Bal-Tec HPM 010. Subsequently, the frozen sample was transferred into a BAF 060 (Leica Microsystems, Vienna, Austria) freeze fracture system where it was fractured with a precooled razor blade. Solvent was allowed to sublime (−80 °C, 30 min). The sample was then transferred to an Ultra 55 cryo-SEM (Zeiss) using the VCT 100 cryo-transfer holder and was imaged at an acceleration voltage of 1–3 kV using the in-lens detector.

### Tensile tests

For the tensile test experiments, all samples were cut into thin strips of 2–3 mm in width, thickness of 30–50 μm, and length of ∼20 mm and measured with an Instron Model 5965 Materials Testing System, equipped with a 50 kN load cell. The deformation rate was 0.2 mm/min. At least 10 specimens of each type were tested. The dimensions of the film were measured using caliber and micrometer.

**Bath Sonication** was performed using MRC Ultrasonic Cleaner D80H. Operation frequency was 43 kHz, and power 80 W.

**Thermogravimetric Analysis (TGA)** experiments were conducted using the thermal analyzer SDT Q 600 (TA Instruments), under air flow (100 mL/min) with a heating rate of 20 °C/min. Samples were measured in alumina pans.

### Differential Scanning Calorimetry (DSC)

Experiments were conducted using a DSC Q200 (TA Instruments), under a N_2_ flow (1 mL/min) with a heating rate of 10 °C/min. Samples were measured in aluminum T-Zero pans.

### Powder X-ray Diffraction (pXRD)

Temperature controlled measurements were made in an Ultima III theta–theta diffractometer (Rigaku, Japan) equipped with a sealed copper anode X-ray tube operating at 40 kV and 40 mA. A bent graphite monochromator and a scintillation detector were aligned in the diffracted beam. θ/2θ scans were performed under specular conditions in Bragg–Brentano geometry with variable slits. The test sample was placed in a medium/low temperature attachment (Rigaku), and its temperature was controlled by a PTC-30 programmable temperature controller. Phase analysis was performed using the Jade Pro software (MDI, icdd.com).

### Raman Spectroscopy

Raman scattering measurements were performed in backscattering mode using a LabRAM HR Evolution (Horiba, France) confocal microspectrometer using 532 nm excitation. The maximum incident power on the sample was 1–2 mW with a submicrometer spot size. The Raman spectra were collected by a 1024 × 256 pixel open electrode front-illuminated CCD camera (Syncerity, Horiba, x ur home, USA) cooled to −60 °C. The spectra were baseline corrected.

### Preparation of Tyr nanocrystals

Tyr (20 mg) was dissolved in boiling DDW (20 mL) for 30 min until the solution was clear, and the solution was filtered through a poly(ether sulfone) (PES) syringe filter (0.22 μm). The filtered solution was sonicated in a bath sonicator for 3 min, until turbidity was observed. The vial was placed under ambient conditions to allow the precipitation of fibrous Tyr crystals.

### Preparation of composites

*Tyr/HEC*. Hydroxyethyl cellulose (100 kDa, 100 mg) was dissolved in 6 mL of water and stirred for 48 h.

Tyr (20–50 mg) was dissolved in boiling DDW (14 mL) until the solution was clear, and then the solution was filtered through a poly(ether sulfone) (PES) syringe filter (0.22 μm).

The hot Tyr solution was added to the HEC solution, and the mixture was stirred at rt for 12 h to allow the crystallization of Tyr. The mature hybrid was either air-dried in molds having a rectangular (20 cm × 10 cm) or circular (diameter of 10 cm) shape. The resulting film was manually detached.

*Neat HEC film preparation*. A dispersion of HEC (100 mg in 20 mL of water) was placed in a form and then dried in ambient conditions to yield a film of neat HEC

*PCL/HEC/Tyr*. Tyr/HEC hybrid films were placed between two PCL films (50 mg each) in a sandwich-like configuration; the triple composite was sealed by heat-press at 80 °C to yield a PCL/HEC/Tyr hybrid film with the HEC/Tyr encapsulated between the PCL layers.

*Neat PCL film preparation*. PCL (50 mg) was dissolved in CHCl_3_, and after PCL was fully dissolved, Dimethylformamide (DMF) was added to the solution. The solvents were slowly evaporated on a hot plate (100 °C) using a custom-designed Teflon plate. After solvent evaporation, the PCL film was manually detached from the support.

### Biodegradability test

Biodegradability was evaluated according to the standard ISO 17556 procedure at *IMI TAMI Institute for Research& Development Ltd.*, Haifa Bay 2611101, Israel. The samples (films) were ground in a cariogenic mill to result in 300–600 μm particles. The test involved 1.25 g of the polymers (each experiment was duplicated) in the 2 L bioreactors that contained 200 g of inoculum concentrate + vermiculite, activated for a week at 28 ± 1 °C. Vessels with NaOH solution were used for CO_2_ adsorption. Samples for determination of the CO_2_ content were taken on a weekly basis. The test was performed for 149 days. The blank treatments (duplicates, no biodegrading medium) produced an amount of ∼20% CO_2_ compared to the samples in the inoculum, as required in ISO 17556.

## Supporting Information Available

TGA, Raman, DSC, XRD data. Agarose/Tyr data. PCL/HEC/Tyr composite data. Additional SEM images. (PDF)
