# Supplementary material for: Emergent Self-Assembly of Sustainable Plastics Based on Amino Acid Nanocrystals
Source: ACS Nano. 2023 Oct 23;17(21):20962–7. doi: 10.1021/acsnano.3c02528 (PMC10655173; doi:10.1021/acsnano.3c02528)
Supplement: Supplementary file 1 — nn3c02528_si_001.pdf [file nn3c02528_si_001.pdf]

Supplementary information

Emergent self-assembly of sustainable plastics based on amino acid nanocrystals

Angelica Niazov-Elkan,[†] Haim Weissman,[†] Eyal Shimoni,[‡] XiaoMeng Sui,[†,‡] Yishay Feldman,[‡] H. Daniel Wagner,[†] Boris Rybtchinski[†*]

[†]Department of Molecular Chemistry and Materials Science, Weizmann Institute of Science, Rehovot 76100, Israel

[‡]Department of Chemical Research Support, Weizmann Institute of Science, Rehovot 76100, Israel

E-mail: Boris.Rybtchinski@weizmann.ac.il

Agarose/Tyr composite

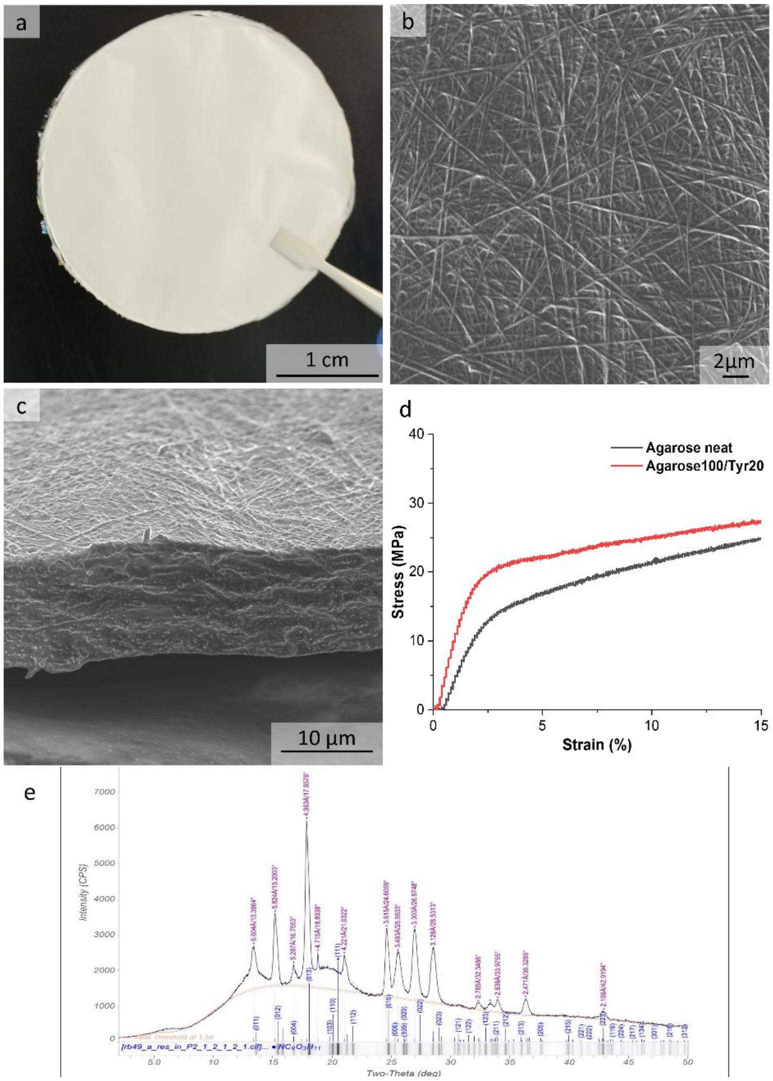

Figure S1. (a) Photograph of an Agarose/Tyr composite film. (b-c) SEM images of (b) of Agarose/Tyr hybrid (top view), the crystalline needles of Tyr show uniform distribution; (c) Cross-section of the composite film. The Tyr crystals are evenly distributed throughout the polymer matrix; (d) Characteristic stress-strain curve of pristine agarose film (black) and Agarose/Tyr=10/2 (wt%) composite (red); (e) XRD diffractogram of the Agarose/Tyr hybrid, showing characteristic Tyr peaks and an amorphous Agarose matrix

TGA, Raman, DSC, XRD data

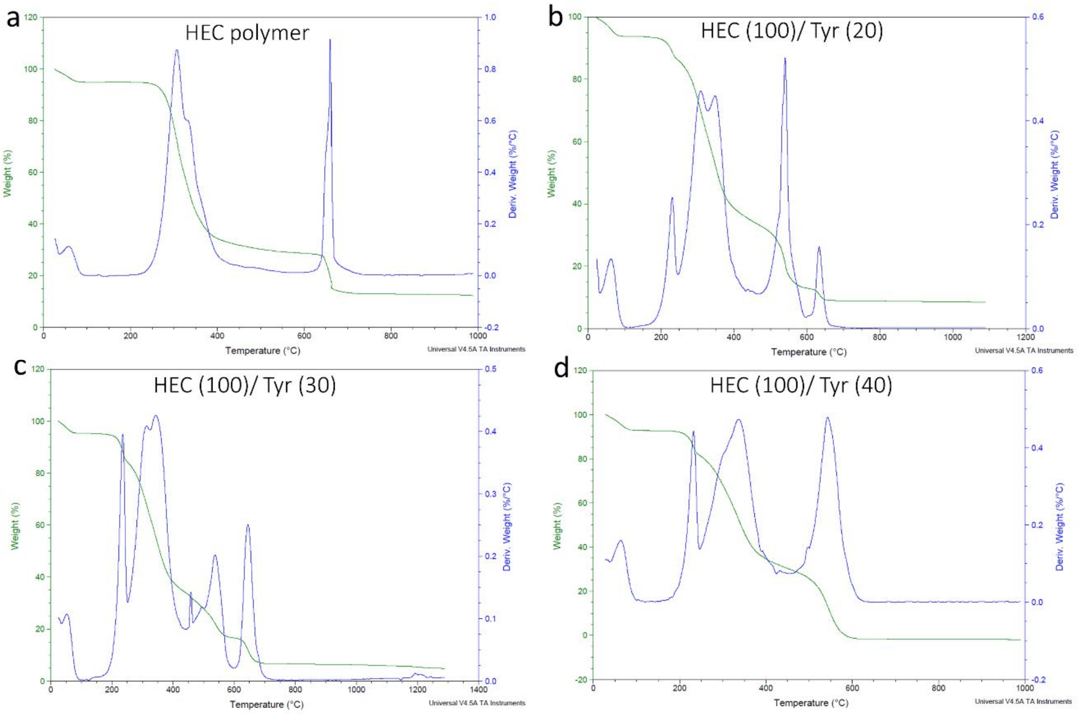

Figure S2. TGA Analysis (a) HEC polymer. (b-d) HEC/Tyr: (b) 10:2 (w/w); (c) 10:3 (w/w); (d) 10:4 (w/w).

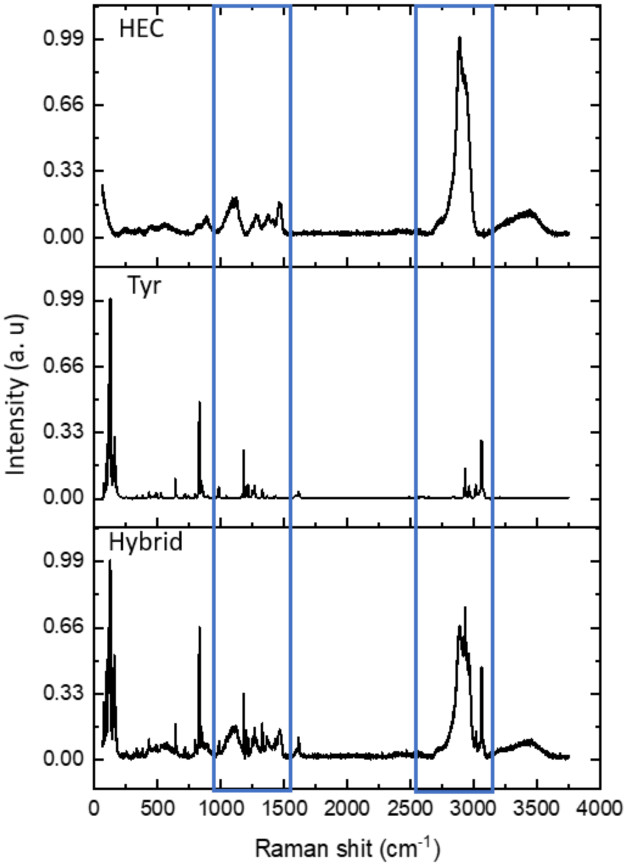

Figure S3. Raman scattering spectra: Films of pristine HEC (top), Tyrosine crystals (middle) and HEC/Tyr hybrids (10:3 wt%), bottom.

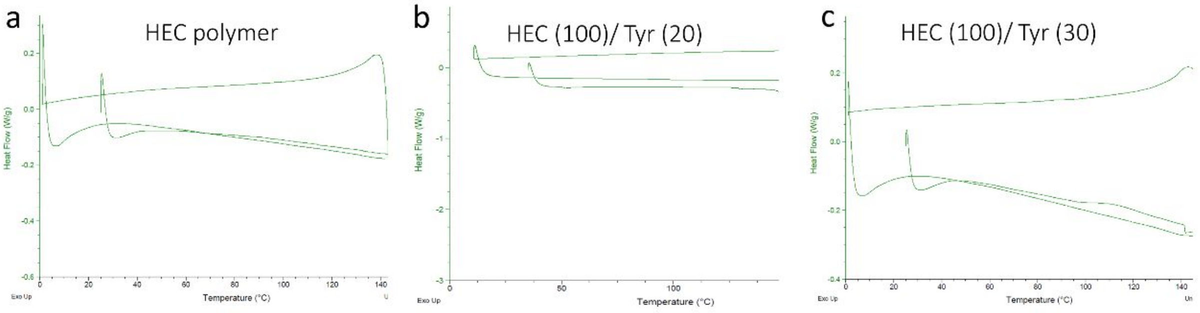

Figure S4. DSC Heat/Cool/Heat Analysis of the HEC/Tyr hybrid. (a) HEC polymer. (b) HEC/Tyr; 100/20 mg. (c) HEC/Tyr; 100/30 mg. No phase transition is observed upon heating up to 150°C

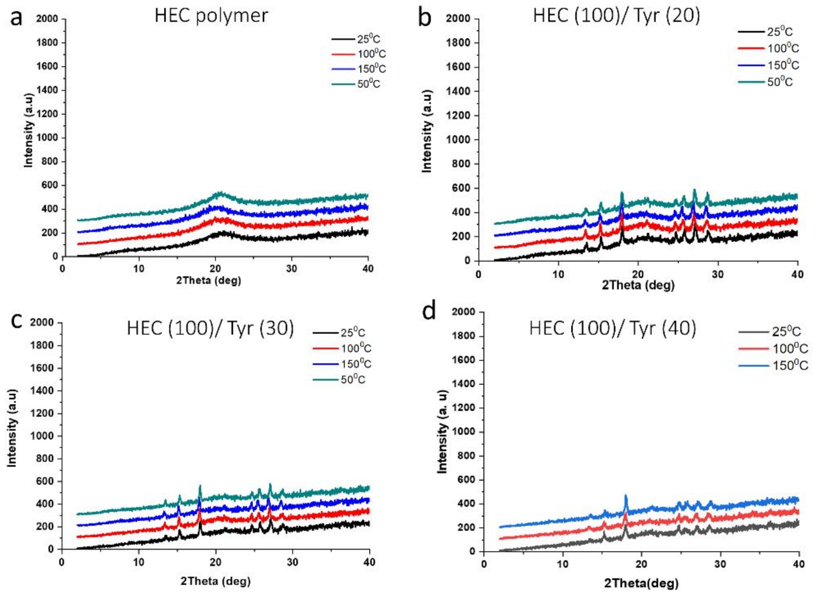

Figure S5. XRD Analysis at different temperatures. (a) HEC polymer. (b) HEC/Tyr; 100/20mg. (c) HEC/Tyr; 100/30mg. (d) HEC/Tyr; 100/40mg.

Tyr high loading composite

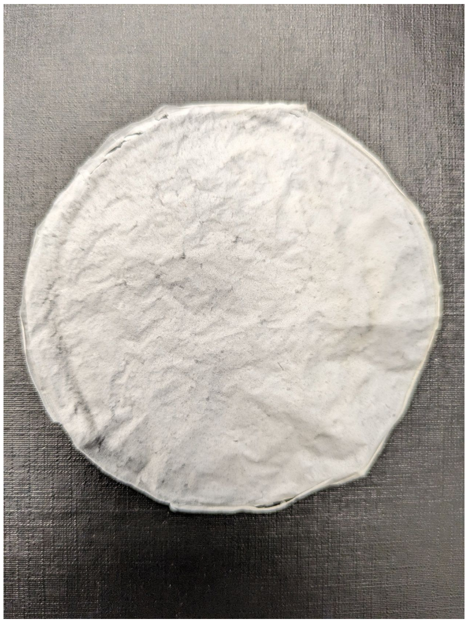

Figure S6. Photograph of a HEC:Tyr=10:5 (wt%) composite (10 cm in diameter), showing defects (darker areas, where less Tyr is present).

Properties of biodegradable plastics

	Elastic modulus (MPa)	Strength (MPa)	Strain (%)	Ref
**PLA**	350-3500	21-60	2.5-6	[1]
**PGA**	6000-7000	60-100	1.5-20	[2]
**(S)-PLA**	1140-2700	15-150	3-10	[3]
**Racemic PLA**	1000-3450	27.5-50	2-10	[4]
**PLA/PGA**	1000-4340	41-55	2-10	[5]
**Chitosan**	135.6	32.9	54.6	[6]
**TPS/Polystyrene**	190-320	8.9-9.5	6-6.8	[7]
**TPS/LDPE**	380-500	10-12	6-37	[8]
**TPS/bacterial cellulose**	361	31	5.3	[9]
**PHB**	3500-4000	40	5-8	[10]
**HEC/Tyr**	830-2090.3	45.9-100.6	25.6-55.2	Current

Table S1. Mechanical properties for biopolymers and blends that are used today for packaging applications.

PCL/HEC/Tyr composite

The susceptibility to water was examined by immersing the films in water for 24 hours, subsequent drying in ambient condition. and further mechanical analysis. No visible morphological change occurred after the water susceptibility test (Figure S5). Tensile test results following immersion in water and drying are presented in Tables S2-S3.

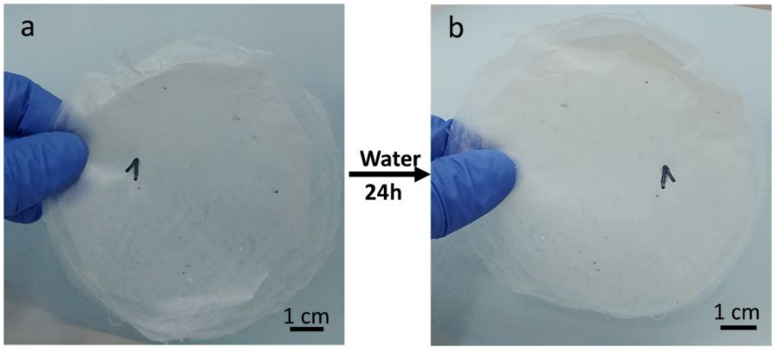

Figure S7. Photograph of PCL/HEC/Tyr composites: (a) Ambient conditions. (b) After immersing the film in water for 24h.

**Sample**	**Elastic Modulus (MPa)**	**Strength (MPa)**	**Strain (%)**	**Toughness (MPa)**
PCL 50mg	224±32	33±22	143±66	29±8
PCL/HEC 5:10	562±153	16±6	21±10	2±1
PCL/HEC /Tyr 5:10:2	829±110	17±2	10±3	1.3±0.6
PCL/HEC /Tyr 5:10:3	1230±197	30±4	13±2	3±1
PCL/HEC /Tyr 5:10:4	1278±155	28±3	8±2	1.6±0.6
PCL/HEC /Tyr 5:10:5	1326±163	24±3	8±2	1.4±0.5

Table S2. The mechanical properties of films made of pristine PCL, PCL/HEC composite and PCL/HEC/Tyr composites. In all cases two 50-mg PCL layers are employed for embedding. The amounts of HEC and Tyr are indicated in the table.

	Elastic Modulus (MPa)	Strength (MPa)	Strain (%)	Toughness (MPa)
PCL/HEC/Tyr 1	662±72	17±2	28±9	3±1
PCL/HEC/Tyr 2	967±221	27±4	27±8	5 ±2

Table S3. Tensile properties of HEC/Tyr Hybrid (PCL/[HEC/Tyr]/PCL = 5:10:2 wt% and 5:10:3 wt% for the composites 1 and 2 respectively after immersing the film in water for 24 h and subsequent drying.

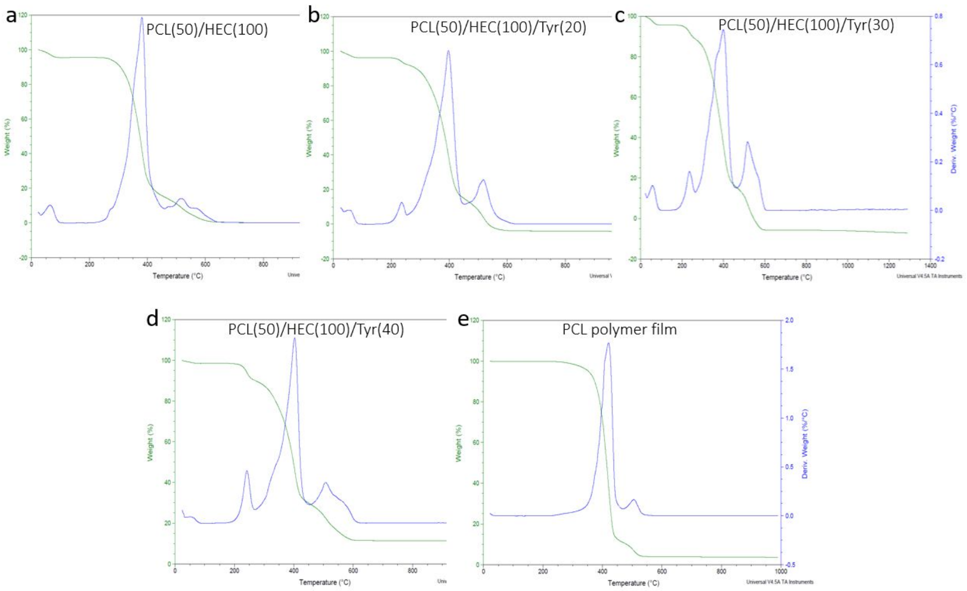

Figure S8. TGA Analysis (a) PCL/HEC polymer. (b-d) PCL/HEC/Tyr; (b) 5:10:2 (w/w); (c) 5:10:3 (w/w); (d) 5:10:4 (w/w). (e) Pristine PCL film .

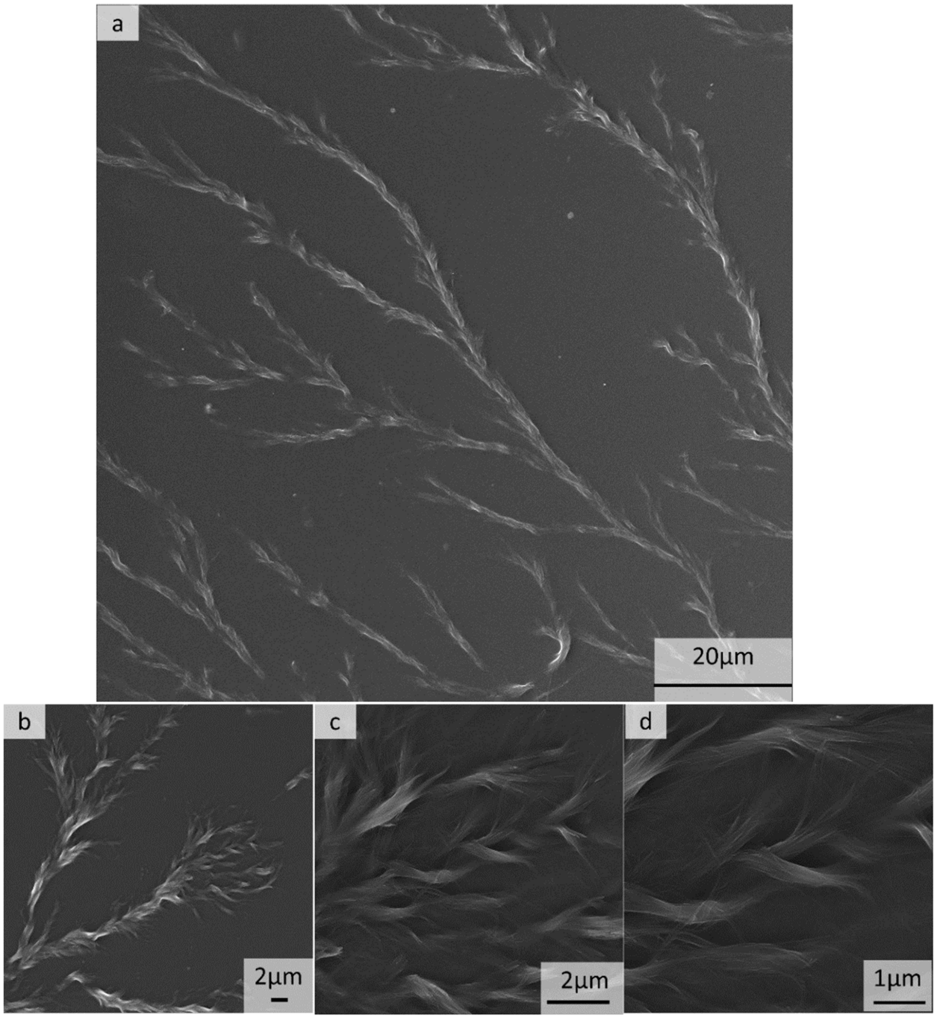

Figure S9. SEM image of a freshly prepared HEC/Tyr hybrid. (a) Characteristic initial structure. (b)-(d) detailed view on the hybrid morphology.

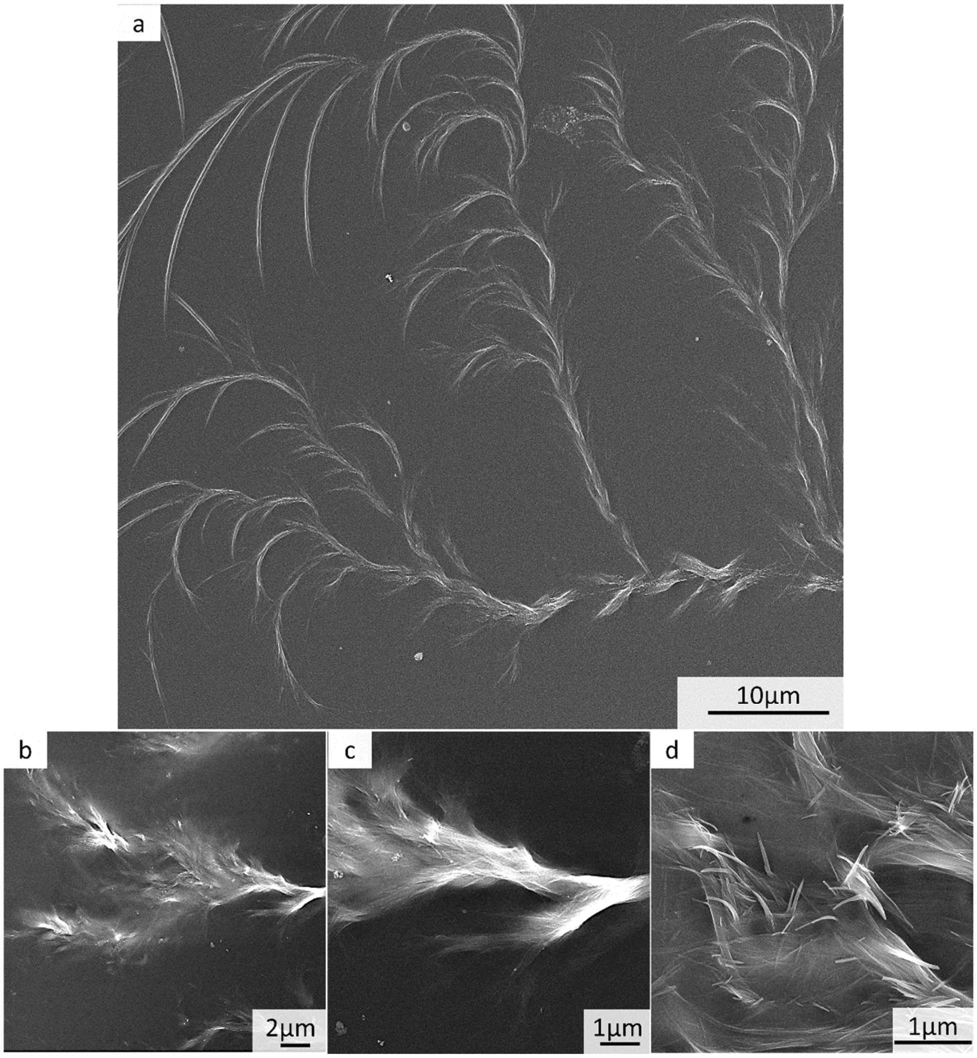

Figure S10. SEM image of a HEC/Tyr hybrid 30 min after preparation. (a) Characteristic structure. Evidently, the growth of the crystals propagates from the initial stems that are observed at time 0. (b)-(d) detailed view of the hybrid morphology.

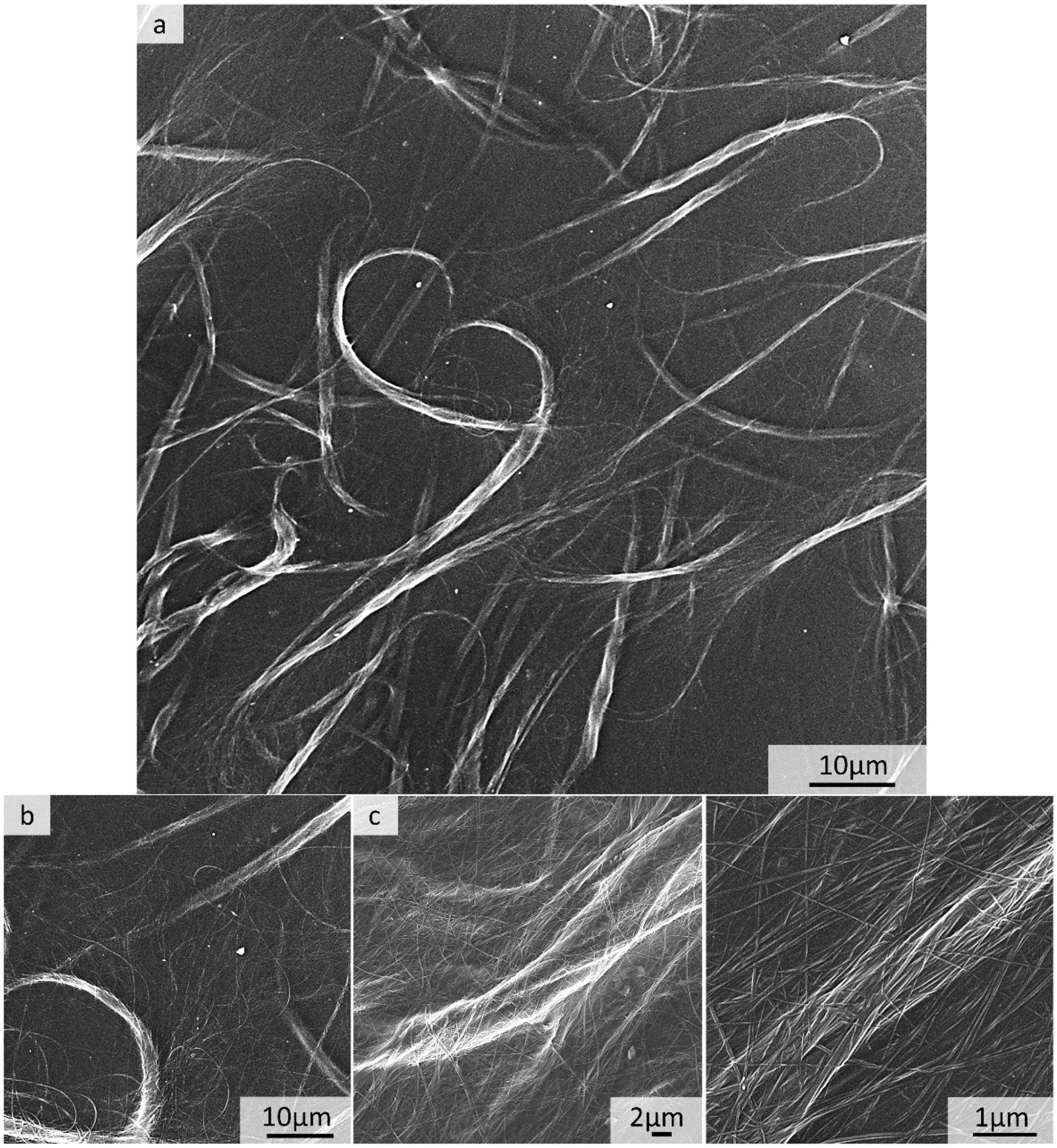

Figure S11. SEM image of HEC/Tyr hybrid 60 min after preparation. (a) Characteristic structure of the developed crystalline fibers from the hybrid bundles (b)-(d) detailed view of the hybrid morphology.

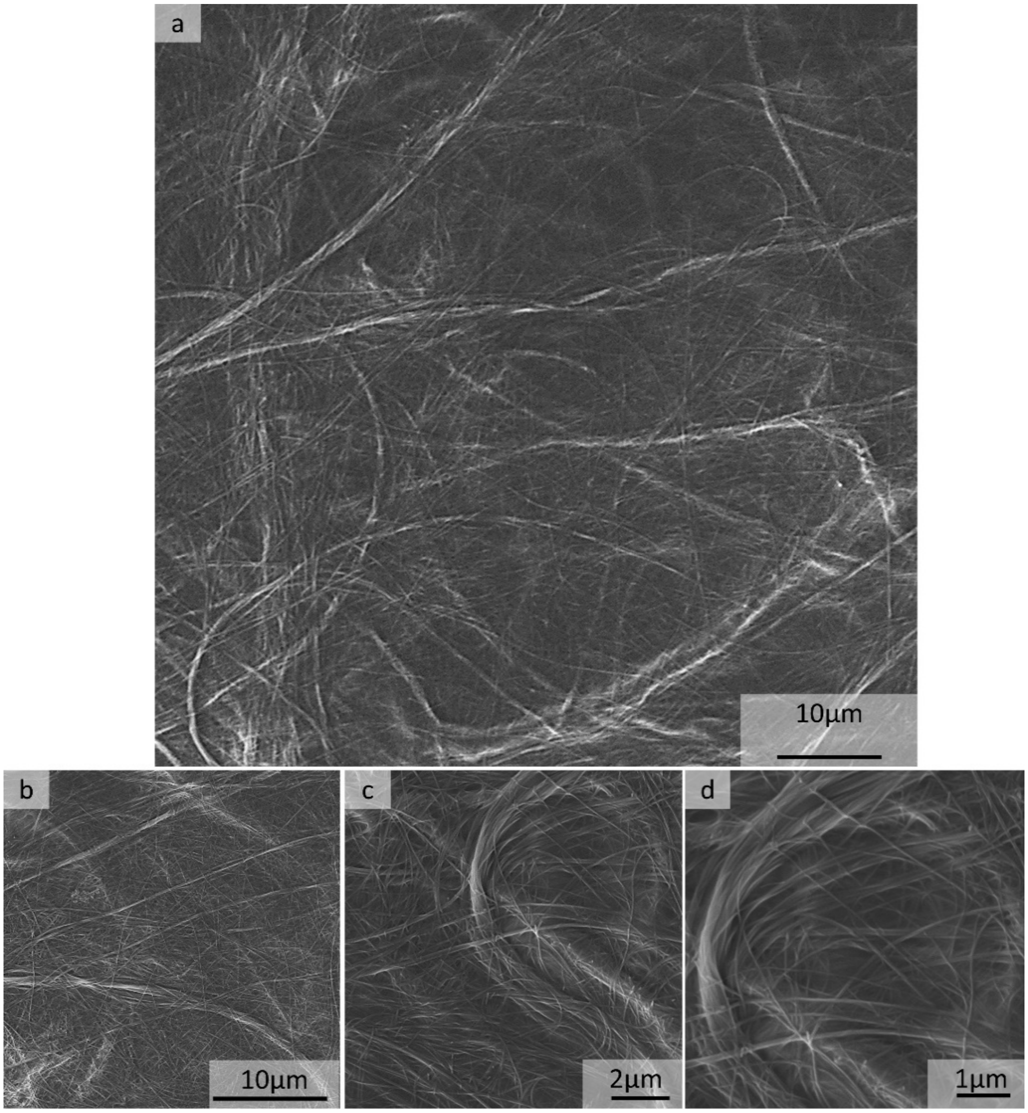

Figure S12. SEM image of HEC/Tyr hybrid 90 min after preparation. (a) Characteristic structure of the mature crystalline fibers with high aspect ratio. (b)-(d) detailed view of the hybrid morphology.

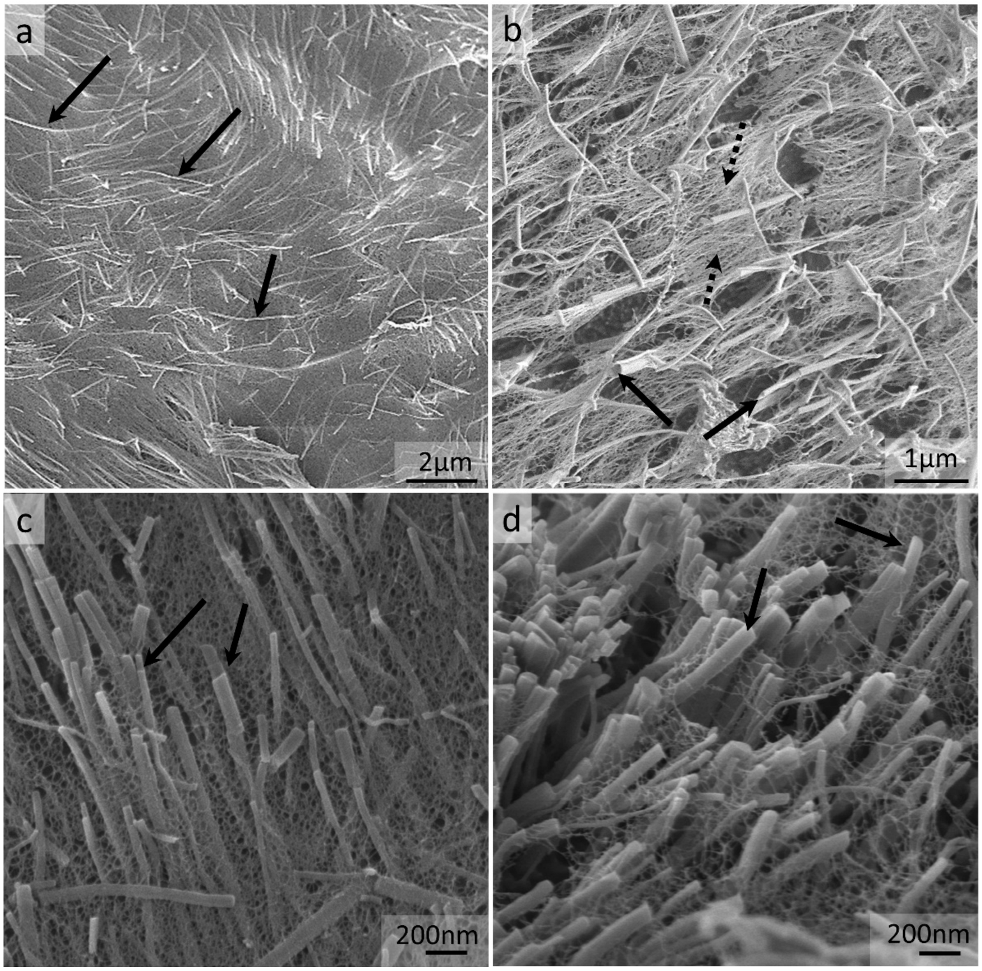

Figure S13. Cryo-SEM image mature HEC/Tyr hybrid, before final water evaporation. (a) Top view of the composite film. (b) Cross-section of the composite film, the polymer matrix is marked with dashed arrows, while the crystals are marked with black arrows. (c) Zoom in to (a) top view, Tyr fibers (marked in arrow) are wrapped by HEC polymer matrix (lacelike structure). (d) Tyr fibers (black arrow) interconnected by the polymer matrix.

Tensile failure imaging, HEC/Tyr (10:3, w/w) and neat HEC

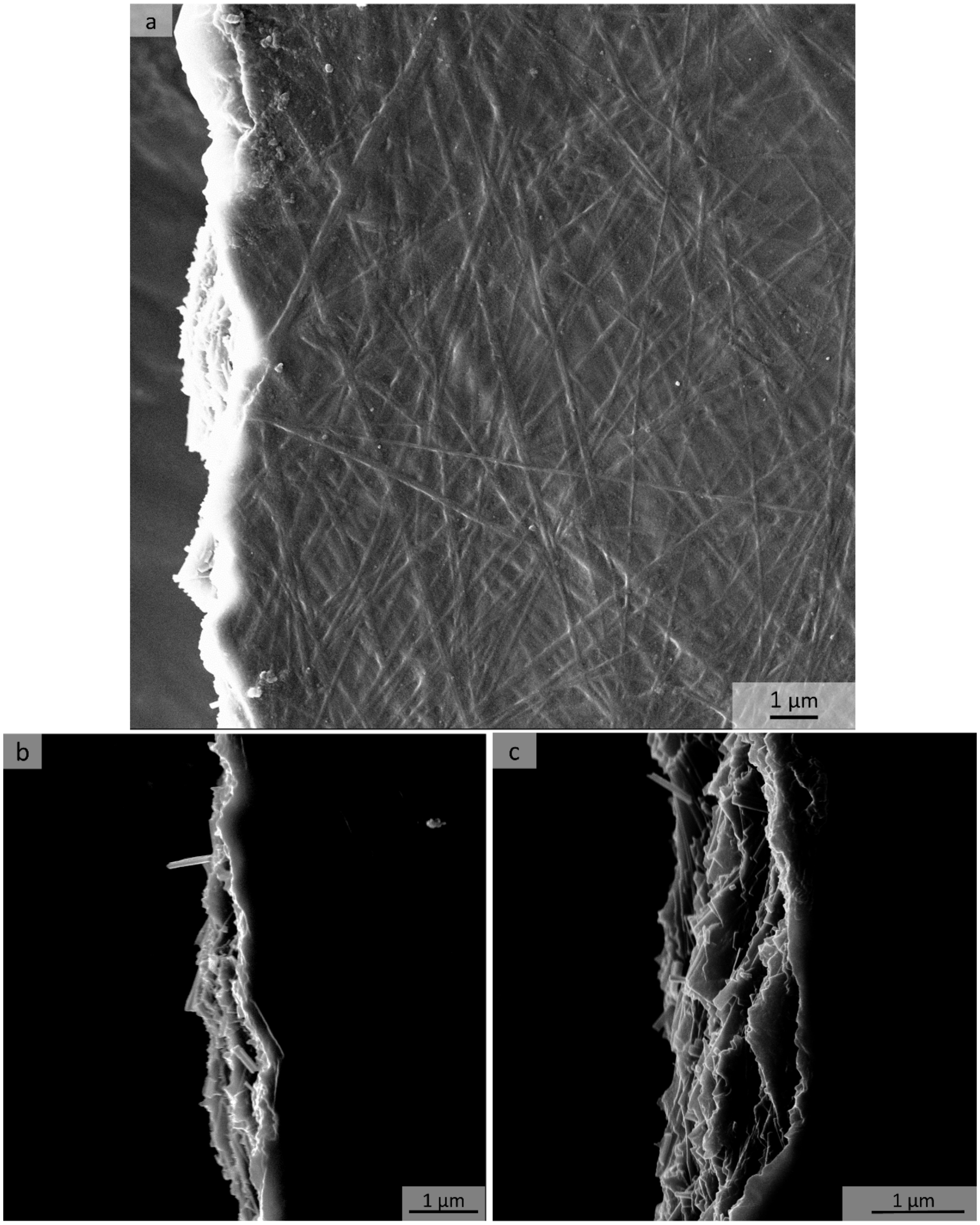

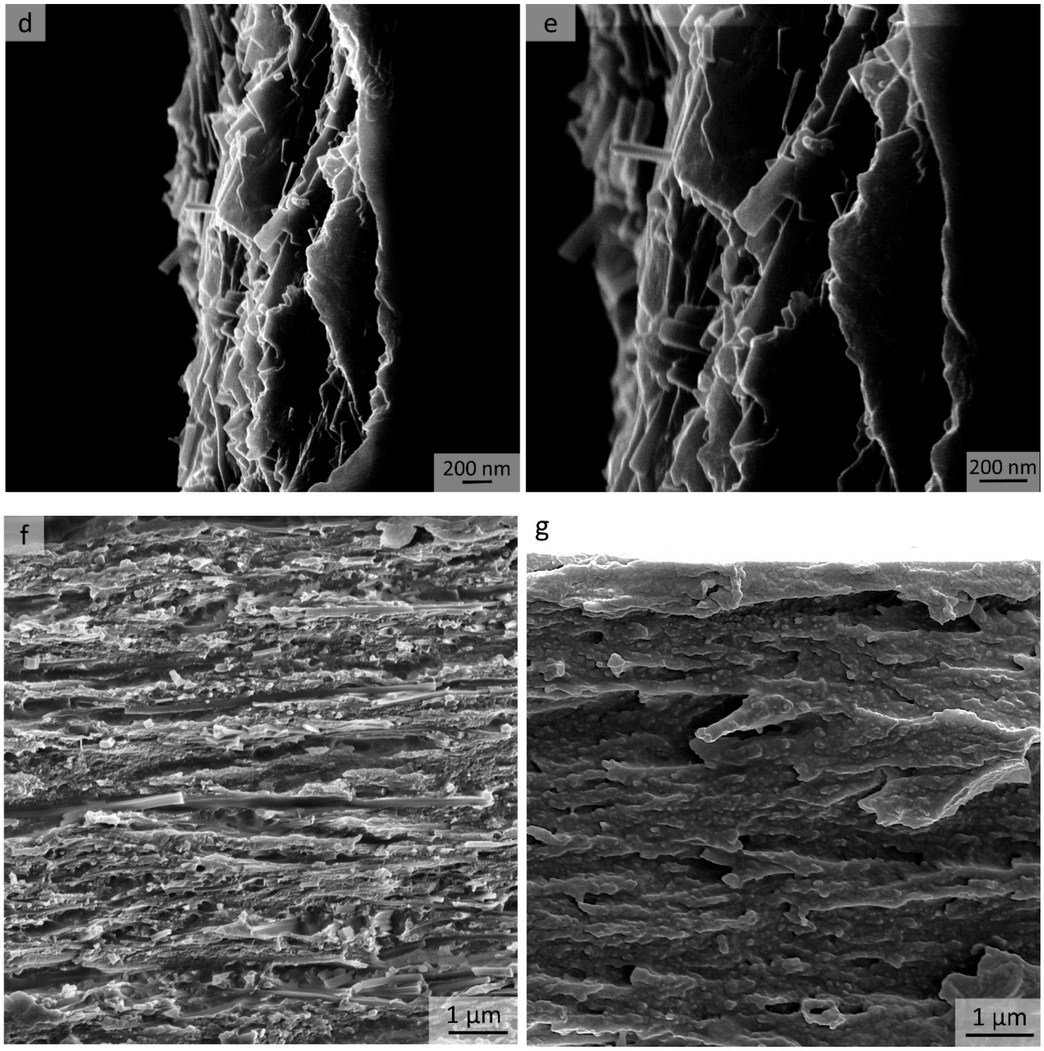

Figure S14. SEM images of the hybrid films following tensile failure test. (a)-(f) SEM images of the failure cross-section of HEC/Tyr hybrid. The failure mechanism involves breaking of the Tyr crystals and rapture of HEC simultaneously as evident from the sharp rupture pattern of the HEC polymer; (g) Cross section of the neat HEC film after failure.
